# 

**Published:** 1908-03-07

**Authors:** 


CONTENTS.

| | PAGE |
|---|---|
| **Clean Milk or Pasteurised Milk** ... ... ... ... | 591 |
| **The Diseases of Historical Characters** ... ... ... | 592 |
| **Annotations—** | |
| Viewing the Body—What are Spirits?—Hospital Appointments and the M.R.C.S. Diploma—The late Mr. Reginald Harrison ... ... ... ... ... ... ... | 593 |
| **Medical Opinion and Movement** ... ... ... ... | 594 |
| **Hospital Clinics—** | |
| Valvular Murmurs in Relation to the Treatment of Cardiac Disease. By R. W. Philip, M.A., M.D., F.R.C.P.E., F.R.S.E. | 597 |
| **Medicine—** | |
| The Relation of Post Diphtheritic Paralysis to Antitoxin ... | 601 |
| Posterior Basal and Epidemic Cerebro-Spinal Meningitis ... | 602 |
| **Points in Surgery—** | |
| Movable Kidney—I. ... ... ... ... ... ... | 603 |
| Some Diseases of the Male Genital System ... ... ... | 604 |
| **Laryngology and Rhinology—** | |
| Lupus of the Upper Air Passages ... ... ... ... ... | 605 |
| **Practical Notes on Diagnosis and Treatment** ... | 606 |
| **Public Health and Hygiene—** | |
| An Interesting Outbreak of Typhoid Fever ... ... ... | 607 |
| **The General Practitioner's Column—** | |
| Chronic Rheumatism and Rheumatoid Arthritis... ... ... | 609 |
| **The Evolution of Medicine—** | |
| Claudius Galenus and His System ... ... ... ... | 610 |
| **Hospital Administration—** | |
| An Impasse at the Royal Victoria Infirmary, Newcastle | 611 |
| Social and Poor-law Problems ... ... ... ... ... | 612 |
| News and Coming Events ... ... ... ... ... | 613 |
| **Nursing Administration—** | |
| Fever Nursing ... ... ... ... ... ... ... | 615 |
| **The Graduates' Medical Schools** ... ... ... | 616 |

